# ERGA-BGE chromosome-level genome assembly of the giant stream lacewing *Osmylus fulvicephalus *(Scopoli, 1763)

**DOI:** 10.12688/openreseurope.23457.3

**Published:** 2026-09-09

**Authors:** Marius F. Maurstad, Dominik Kusy, Eivind A. B. Undheim, Kjetill S. Jakobsen, Astrid Böhne, Rita Monteiro, Thomas Marcussen, Torsten H. Struck, Rebekah A. Oomen, Ignas Bunikis, Christian Tellgren-Roth, Julia Heintz, Susana Häggqvist, Linnea Jonsäll, Nina Lager, Tuuli Larva, Mai-Britt Mosbech, Martin Pippel, Olga Vinnere Pettersson, Chiara Bortoluzzi

**Affiliations:** 1 Centre for Ecological and Evolutionary Synthesis, University of Oslo, Oslo, Norway; 2 Czech Advanced Technology and Research Institute (CATRIN), Palacký University Olomouc, Olomouc, Czech Republic; 3 Leibniz Institute for the Analysis of Biodiversity Change, Museum Koenig Bonn, Bonn, Berlin, 53113, Germany; 4 Natural History Museum, University of Oslo, Oslo, Norway; 5 University of Oslo Centre for Ecological and Evolutionary Synthesis, Oslo, Norway; 6 Department of Biological Sciences, University of New Brunswick Saint Joh, Saint John, Canada; 7 Tjärnö Marine Laboratory, University of Gothenburg, Gothenburg, Sweden; 8 Centre for Coastal Research, University of Agder, Kristiansand, Norway; 9 National Genomics Infrastructure, Uppsala Genome Center, SciLifeLab, Uppsala, Box 815, 751 08, Sweden; 10 Department of Immunology, Genetics and Pathology, Uppsala University, Uppsala, Box 815, 751 08, Sweden; 11 SciLifeLab Bioinformatics, National Bioinformatics Infrastructure, Uppsala University, SciLifeLab, Uppsala, Box 3037, 750 03, Sweden; 12 Department of Cell and Molecular Biology, Uppsala University, Uppsala, Box 596, 751 24, Sweden; 13 Swiss Institute of Bioinformatics, Amphipôle, Quartier UNIL-Sorge, Lausanne, Vaud, Switzerland

**Keywords:** Osmylus fulvicephalus, genome assembly, European Reference Genome Atlas, Biodiversity Genomics Europe, Earth Biogenome Project, giant stream lacewing, Osmylidae, Neuroptera

## Abstract

The giant stream lacewing, *Osmylus fulvicephalus* (Scopoli, 1763), is a widespread European species belonging to the insect order Neuroptera. Its cryptic larvae are predators found at the banks of streams and smaller rivers where they use their piercing, lance-shaped stylets to inject venom into their arthropod prey. Here, we present the reference genome of the giant stream lacewing as a crucial resource for uncovering the genetic basis of venom evolution in Neuroptera. The chromosome-level genome encompasses 674.7 Mb and is composed of 60 contigs and 24 scaffolds where 99.2% of the assembly is distributed among the 6 contiguous chromosomal pseudomolecules and two sex chromosomes (X and Y). Contig and scaffold N50 have a value of 51.5 Mb and 116.2 Mb, respectively. This reference genome is the first genomic resource from the family of lance lacewings, providing valuable data for clarifying the phylogenetic placement of the family *Osmylidae* within Neuroptera.

## Introduction

*Osmylus fulvicephalus* is a member of the small family *Osmylidae* (giant lacewings), within the order Neuroptera (net-winged insects). This family comprises relatively few species globally, with *O. fulvicephalus* being the sole representative in Europe ( Kriska, 2023). The species has a broad European distribution, ranging from the British Isles and the Iberian Peninsula in the west, through southern Scandinavia in the north, to the southern Balkans in the southeast. Within the Czech Republic, the species is found predominantly at mid-elevation localities where suitable riparian habitats are present.

*Osmylus fulvicephalus* has not been assessed for the IUCN Red List and is not listed under any international conservation conventions. At the regional level (Czech Republic), the species is locally relatively abundant but absent from many areas. Its populations may be threatened primarily by insensitive regulation of smaller watercourses and water pollution.

**Figure 1.**
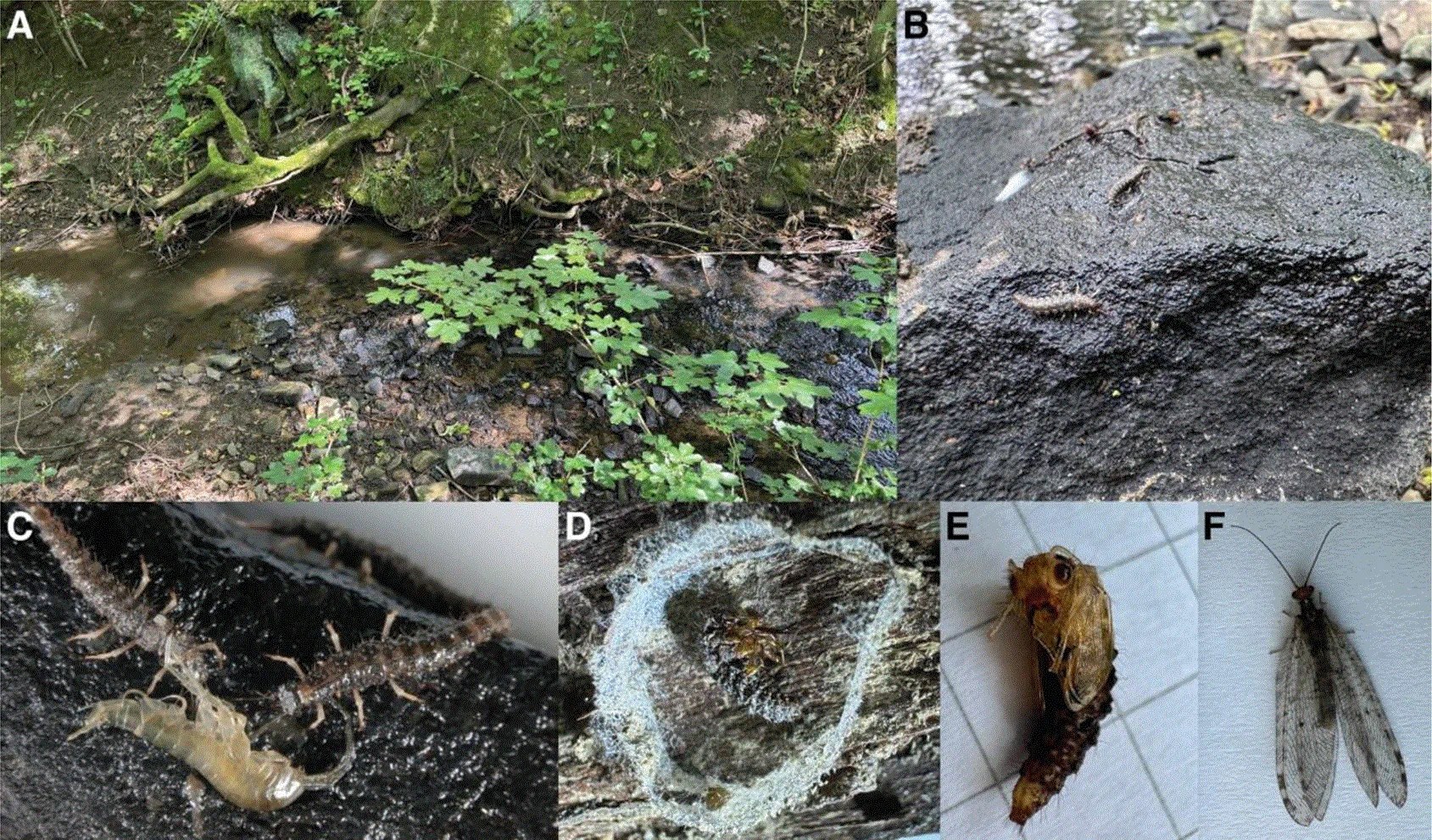
Overview of habitat, feeding strategy, and developmental stages. A) Sampling locality habitat. B) Larvae *in situ* on the underside of a submerged stone. C) Larva feeding on *Gammarus fossarum.* D) Larva constructing a silk chamber prior to pupation. E) Pupa. F) Adult.

*Osmylus fulvicephalus* is closely associated with small to medium-sized watercourses with abundant riparian vegetation ( Miguélez & Valladares, 2008). The predatory larvae inhabit detritus along stream banks and also hunt below the water surface. Adults are active from approximately May to July, resting on nearby vegetation and displaying a slow, fluttering flight when disturbed. The species occasionally exhibits mass aggregation behaviour at specific sites such as shrubs or under bridges ( Elliott, 2009, pers. obs.). Both larvae and adults are predators, with larvae found to be feeding on small arthropods such as mites and collembolans during the first larval stage, while in the second and third stages they mainly feed on larvae of *Chironomidae* and *Tipulidae* ( Elliott, 2009; Miguélez & Valladares, 2008) and *Gammarus fossarum* (pers. obs.) ( Figure 1). In addition, adults also feed on other items such as pollen ( Devetak & Duelli, 2007).

Neuroptera is a venomous insect lineage where the larvae have evolved specialized maxillary stylets through which they inject paralyzing and liquefying venom ( Fischer et al., 2024; Maurstad et al., 2026). The giant stream lacewing represents an early branch of Neuroptera, and therefore its genome will be crucial for uncovering the evolutionary history of the venom system in Neuroptera. Furthermore, the phylogenetic positioning of *Osmylidae* within *Osmyloidea* is still unresolved ( Lai et al., 2025; Vasilikopoulos et al., 2020), which this genomic resource will help address.

The generation of this reference resource was coordinated by the European Reference Genome Atlas (ERGA) initiative’s Biodiversity Genomics Europe (BGE) project, supporting ERGA’s aims of promoting transnational cooperation to promote advances in the application of genomics technologies to protect and restore biodiversity ( Mazzoni et al., 2023).

## Materials & Methods

ERGA’s sequencing strategy includes Oxford Nanopore Technology (ONT) and/or Pacific Biosciences (PacBio) for long-read sequencing, along with Hi-C sequencing for chromosomal architecture, Illumina Paired-End (PE) for polishing (i.e. recommended for ONT-only assemblies), and RNA sequencing for transcriptomic profiling, to facilitate genome assembly and annotation.

### Sample and sampling information

On 9 June 2024, Dominik Kusy sampled multiple larvae and five adult specimens of *Osmylus fulvicephalus.* The five adult specimens were all males. The specimens were identified by Dominik Kusy based on morphology, which for the species is characteristic of the *Osmylidae* family. Larvae were collected among stones along the bank of a small stream in Cvrčovický Potok, Milovice, Czech Republic (49.575420, 17.282286), then transferred to the laboratory and reared until adult emergence, at which point sex was determined. Adults were collected at a nearby site (49.214813, 17.353428) beneath a small concrete bridge spanning the same stream. Individuals were found resting singly on the bridge walls. Following sex determination, specimens were euthanised by being flash-frozen in liquid nitrogen and were preserved at -80 °C until DNA extraction. No permits were required for sampling.

### Vouchering information

No physical reference material for the sequenced specimen has been deposited in a collection, but additional DNA and RNA material can be accessed at Leibniz Institute for the Analysis of Biodiversity Change https://bonn.leibniz-lib.de/en/ under IDs ZFMK-DNA-FD20030308 and ZFMK-RNA-FD20030344. No reference tissue material for the sequenced specimen has been deposited in a biobank. One digital image (e-voucher) of the sequenced individual is available from the ERGA’s EBI BioImageArchive dataset https://www.ebi.ac.uk/biostudies/bioimages/studies/S-BIAD1012?query=ERGA under accession ID SAMEA115755031.

### Genetic information

The estimated genome size, based on ancestral taxa, is 0.49 Gb. This is a diploid genome with a haploid number of 8 chromosomes (2n = 16) ( Challis et al., 2023). The male is the heterogametic sex with XY sex determination system. The closest related species within the same genus has 6 autosomes and XY chromosomes ( Hirai, 1956). All information for this species was retrieved from Genomes on a Tree ( Challis et al., 2023).

### DNA/RNA processing

DNA was extracted from the whole specimen of a single adult male (wings were removed) following PacBio’s instruction described in “Procedure & checklist Extracting HMW DNA from insects using the Nanobind ^®^ PanDNA kit” (REV02 JAN2025) . In short, the sample was crushed on ice using a pellet pestle, followed by CT washes. Lysis and digestion were then performed using Proteinase K and subsequent RNase treatment. The lysate was then filtered, after which the DNA was bound to a Nanobind disk. The disk underwent multiple wash steps before the purified HMW DNA was finally eluted. Quantification was performed using a Qubit dsDNA BR Assay kit (Thermo Fisher Scientific) and integrity was assessed using a FemtoPulse system (Agilent). DNA was stored at 4 °C until usage. The biobank barcode for the adult male is FD20030308.

RNA was extracted from a whole body of single adult female using the TRIzol Reagent and Phasemaker Tubes Complete System (Invitrogen Cat #A33250) following the Invitrogen user guide (Pub. No. MAN0016163 Rev. A.0) except for steps 2.a. and 3.d., which were omitted. RNA was resuspended in 87.5 μl RNase-free water and immediately subjected to DNase treatment followed by purification according to the RNeasy Micro Handbook (pages 74 and 53). Quantity and integrity were assessed using the Agilent 2100 Bioanalyzer instrument. The eluted RNA was stored at −70°C. The Biobank barcode for the adult female is FD20030344.

### Library preparation and sequencing

One PacBio SMRTbell library was constructed from 3 μg DNA according to the PacBio protocol “Preparing whole genome and metagenome libraries using SMRTbell prep kit 3.0” (PN 102–982-300 (Rev 06 Jan 2025)). DNA shearing was done using the Megaruptor 3 (Diagenode), followed by a clean-up step using PacBio cleanup beads. Adapter ligation was performed overnight. Size selection was performed on PippinHT (Sage Science) using the 0.75% 6-10 k HighPass 75e protocol. Primer annealing and polymerase binding were performed using the Revio SPRQ polymerase kit (PN 103–496-900). Finally, one Revio SMRTcell 25 M was sequenced on the PacBio Revio System. Hi-C library was generated from whole body of a single male as described in the User Guide for Library Preparation for Arima High Coverage HiC Kit (A160186 v02) (Arima Genomics) and sequenced on the NovaSeq X Plus system and XLEAP-SBS sequencing chemistry (Illumina Inc.). RNA sequencing library was prepared from 100 ng total RNA using the TruSeq stranded mRNA library preparation kit (cat# 20020595, Illumina Inc.) including polyA selection. Unique dual indexes (cat# 20022371, Illumina Inc.) were used. The library preparation was performed according to the manufacturers’ protocol (#1000000040498). The library was sequenced on Illumina NovaSeq X Plus system on a 25B flow cell using paired-end 150 bp read length and XLEAP-SBS sequencing chemistry. In total 164x HiFi and 49x HiC data were sequenced to generate the assembly.

### Genome assembly methods

The genome was assembled using the Earth Biogenome Project - Genome Assembly Workflow ( https://workflowhub.eu/workflows/1106, git commit: e6e9c1621b). Briefly, reads were randomly subsampled to a targeted 60X read coverage from the total dataset (164x) using a custom Python script that sequentially collected reads until the target sequence yield was achieved. Reads were then filtered for a minimum length of 1,000 bp, with no additional filtering or weighting applied for read quality. Initial contigs were assembled using hifiasm v0.25.0-r726, followed by contamination removal with FCS-GX (git commit: v0.5.4–5-g5dfd516). Retained haplotig sequences were annotated with purge_dups v.1.2.6. Due to a low yield of long-range intra-chromosomal interactions in the Hi-C dataset (cis > 1 kb: 3.5%; cis > 40 kb: 2.6%, despite a ~25% duplication rate), automated Hi-C scaffolding was insufficient for resolving long-range chromosomal structure. Consequently, scaffolding was performed via manual curation using the curationpretext pipeline ( https://pipelines.tol.sanger.ac.uk/curationpretext, v1.5.1), PretextView v1.0.5 and the Rapid Curation Toolkit ( https://gitlab.com/wtsi-grit/rapid-curation ). In this step additional information from tiara ( https://github.com/ibe-uw/tiara v1.0.3), a deep-learning-based sequence classifying tool, MitoHifi v3.2.3, BlobtoolKit v4.4.5 and purge_dups were incorporated to facilitate the curation process. A synteny analysis based on GCA_958496175, GCA_958496155, GCF_905475395.1 and GCA_020423425.1 confirmed the sex chromosomes X and Y and the curated scaffold joins of the six autosomes. Finally, the mitochondrial genome was assembled as a single circular contig of 21080 bp using the MitoHifi pipeline based on the reference sequence NC_050653.1 and the subsampled Hifi read set and is available together with the genome assembly under the ENA umbrella project PRJEB91535.

## Results

### Genome assembly

The genome was assembled into one haplotype using Hi-C phasing and was curated to chromosome level ( Tables 1 and 2).

**Table 1.** Genome assembly statistics.

Genome assembly	
Assembly name	inOsmFulv5.1
Assembly accession	GCA_977109365.1
Assembly level	Chromosome
Span (Gb)	0.67 Gb
Number of chromosomes	6 autosomes, 2 sex chromosomes (X, Y)
Number of contigs	60
Contig N50 (bp)	51,542,635
Number of scaffolds	24
Longest scaffold length (bp)	128,807,968
Scaffold N50 (bp)	116,168,846
Organelles (bp)	Mitochondrion (21,080)

**Table 2.** Chromosomal pseudomolecules in the genome assembly of *Osmylus fulvicephalus*, inOsmFulv5.1 reference.

INSDC accession	Chromosome	Size (Mb)	GC%
OZ372949.1	1	128.80	36.0
OZ372950.1	2	125.49	36.0
OZ372951.1	3	116.16	36.0
OZ372952.1	4	93.17	37.0
OZ372953.1	5	81.86	36.5
OZ372954.1	6	79.51	36.5
OZ372955.1	X	34.67	35.5
OZ372956.1	Y	9.62	39.0
OZ372948.1	MT	0.21	-

**Figure 2.**
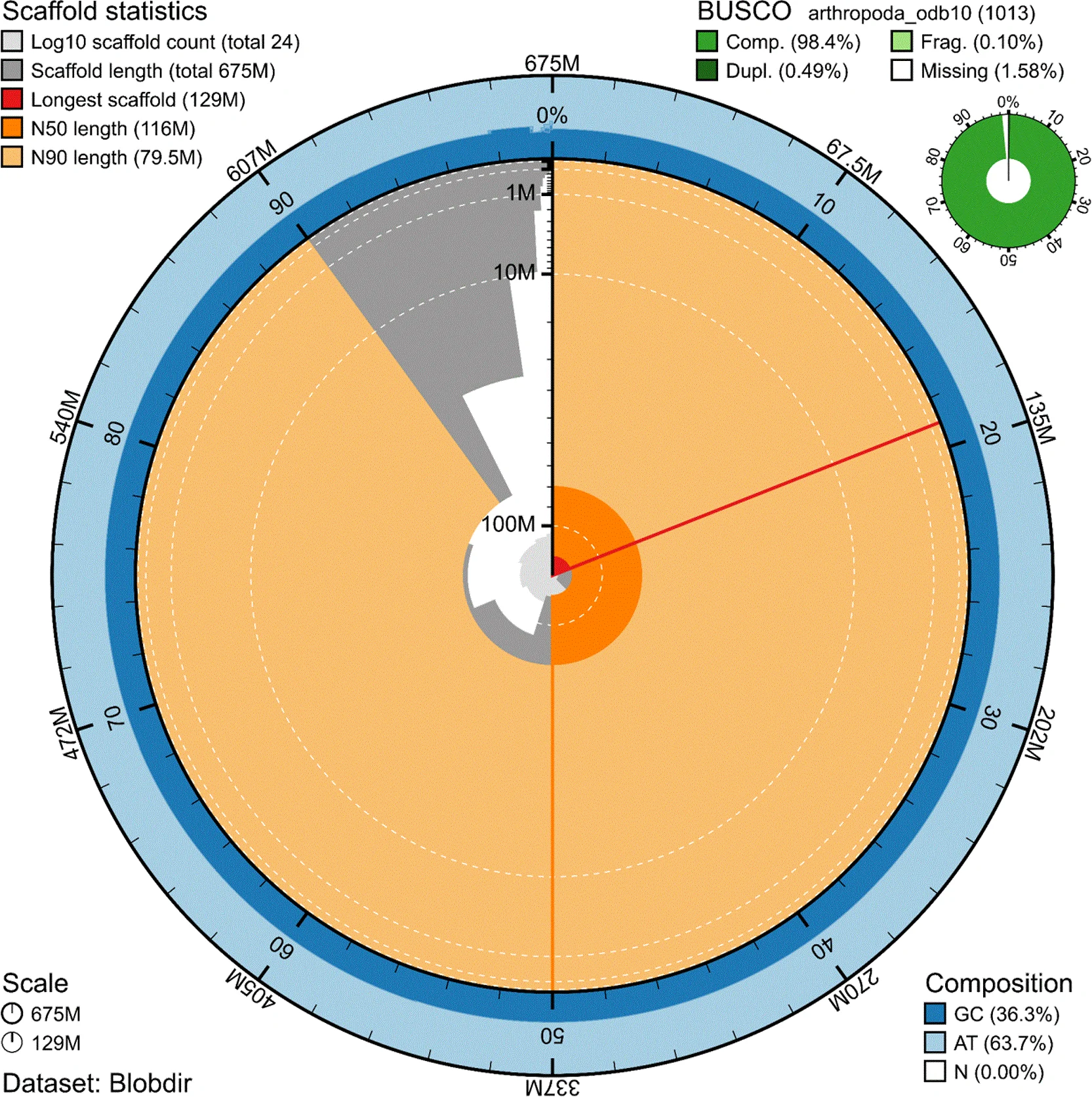
Snail plot summary of assembly statistics. The main plot is divided into 1,000 size-ordered bins around the circumference, with each bin representing 0.1% of the 674,668,284 bp assembly including the mitochondrial genome. The distribution of sequence lengths is shown in dark grey, with the plot radius scaled to the longest sequence present in the assembly (129 Mb, shown in red). Orange and pale-orange arcs show the scaffold N50 and N90 sequence lengths (116,168,846 and 79,514,024 bp), respectively. The pale grey spiral shows the cumulative sequence count on a log-scale, with white scale lines showing successive orders of magnitude. The blue and pale-blue area around the outside of the plot shows the distribution of GC, AT, and N percentages in the same bins as the inner plot. A summary of complete, fragmented, duplicated, and missing BUSCO genes found in the assembled genome from the Arthropoda database (odb10) is shown in the top right.

**Figure 3.**
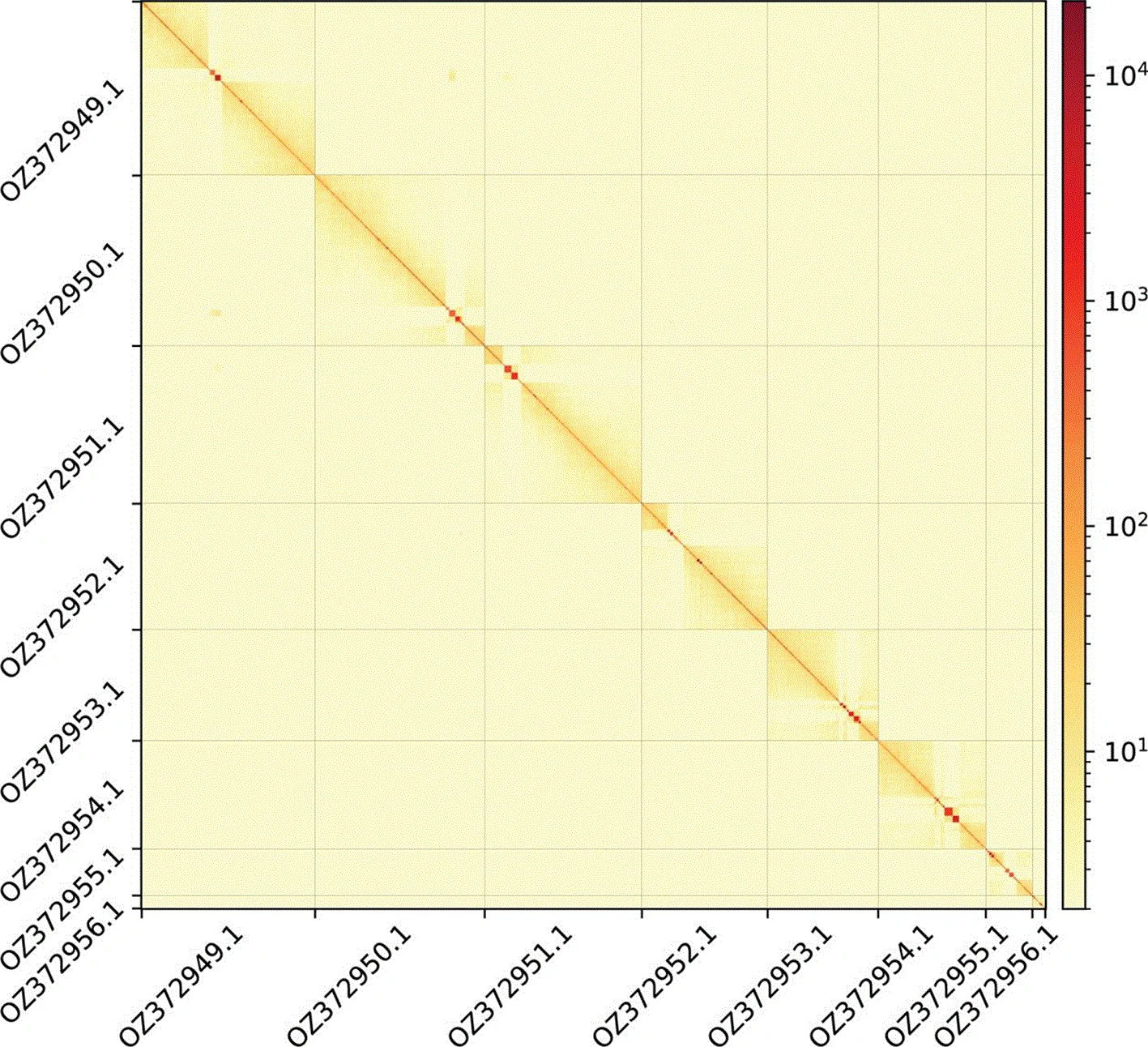
Hi-C contact map showing spatial interactions between regions of the genome. The diagonal corresponds to intra-chromosomal contacts, depicting chromosome boundaries. The frequency of contacts is shown on a logarithmic heatmap scale. Hi-C matrix bins were merged into a 200 kb bin size for plotting.

The genome assembly has a total length of 674,668,284 bp in 24 scaffolds including the mitogenome ( Figures 2 and 3), with a GC content of 36.3%. The assembly has a contig N50 of 51,542,635 bp and L50 of 5 and a scaffold N50 of 116,168,846 bp and L50 of 3. The assembly has a total of 36 gaps, totalling 7.2 kb in cumulative size. The single-copy gene content analysis using the Arthropoda database (odb10) with BUSCO ( Manni et al., 2021) resulted in 98.4% completeness (97.9% single and 0.5% duplicated). 87.8% of reads k-mers were present in the assembly and the assembly has a base accuracy Quality Value (QV) of 68.5 as calculated by Merqury ( Rhie et al., 2020).

## Author contributions

DK collected the species; DK identified the species; DK and MFM sampled and preserved biological material and provided metadata; AsB, RM, TM, THS, RAO, DK, MFM, EABU, and KSJ provided support in sampling, shipping of biological material, metadata collection, and management; JH and M-BM extracted DNA and RNA; NL and LJ performed HiFi library preparation and sequencing under supervision of SH and coordination of OVP coordinated; CTR and IB performed raw data processing and data QC; TL prepared Hi-C DNA and library; MP performed genome assembly and curation; CB generated the analysis; CB, DK and MFM drafted the report. All authors contributed to the writing, review, and editing of this genome note and read and approved the final version.

## Data Availability

*Osmylus fulvicephalus* and the related genomic study were assigned to Tree of Life ID (ToLID) ‘inOsmFulv5’ and all sample, sequence, and assembly information are available under the umbrella BioProject PRJEB91535. The sample information is available at the following BioSample accessions: SAMEA115755033 and SAMEA115755034. The genome assembly is accessible from ENA under accession number GCA_977109365.1 and the annotated genome will be made available through the Ensembl website ( https://projects.ensembl.org/erga-bge /). Sequencing data produced as part of this project are available from ENA at the following accessions: ERX14574817 and ERX14538627. Documentation related to the genome assembly and curation can be found in the ERGA Assembly Report (EAR) document available at https://github.com/ERGA-consortium/EARs/tree/main/Assembly_Reports/Osmylus_fulvicephalus/inOsmFulv5. Further details and data about the project are hosted on the ERGA portal at https://portal.erga-biodiversity.eu/data_portal/446453.
