# Supplementary material for: ERGA-BGE chromosome-level genome assembly of the giant stream lacewing  Osmylus fulvicephalus (Scopoli, 1763)
Source: Open Res Eur. 2026 Sep 9;6:136. Originally published 2026 Apr 28. [Version 3] doi: 10.12688/openreseurope.23457.3 (PMC13554741; doi:10.12688/openreseurope.23457.3)
Supplement: Peer review history

# Reviewer report: Reviewer response for version 2

**DOI:** 10.21956/openreseurope.26149.r76832

**Published:** 2026-08-05

**Authors:** Annabel Whibley

**Affiliations:** 1 Bragato Research Institute, Clyde, New Zealand

Maurstad and colleagues report the reference genome assembly of the giant stream lacewing *Osmylus fulvicephalus. *The concise reports contains most essential metadata, data accessions links and sufficient descriptions of the methods (see below for missing organelle details). The natural history descriptions are nicely written. The assembly is of high quality, with good completeness, accuracy and contiguity stats.

 I have some further comments on some of the methodological details:

 Was DNA-barcoding used to verify the species identity of the specimen?

***“*** Briefly, reads were subsampled to 60X read coverage and initial contigs were assembled using hifiasm v0.25.0-r726”. It would be helpful to know how you performed this subsampling (from 164x to 60x). Was this a randomly-sampled proportion of the total dataset or did you provide any weighting to quality or length parameters?

 “Retained haplotig sequences were annotated with purge_dups v.1.2.6. Due to suboptimal HiC quality the scaffolding was done manually in the manual curation step based on the curationpretext pipeline”. The contact map shown does seen quite sparse: was the low number of recovered contacts the issue with the dataset, can you be any more specific about the “suboptimality”?

 Why not report the mitochondrial assembly in Tables 1 and 2?

***Minor***

 Abstract: for a genome of this size, reporting the total assembly length in Mb rather than Gb would be more usual.

 “In this step additional information from tiara ( https://github.com/ibe-uw/tiara v1.0.3), a deep-learning-based sequence classifying tool, MitoHifi v3.2.3, BlobtoolKit v4.4.5 and purge_dups **was **incorporated to facilitate the curation process” In this line, “was” should be “were”.

Are sufficient details of methods and materials provided to allow replication by others?

Partly

Is the rationale for creating the dataset(s) clearly described?

Yes

Are the datasets clearly presented in a useable and accessible format?

Yes

Are the protocols appropriate and is the work technically sound?

Yes

Reviewer Expertise:

NA

I confirm that I have read this submission and believe that I have an appropriate level of expertise to confirm that it is of an acceptable scientific standard, however I have significant reservations, as outlined above.

# Response

**Published:** 2026-08-31

**Authors:** Chiara Bortoluzzi

**Affiliations:** Environmental Bioinformatics Group, Swiss Institute of Bioinformatics, Lausanne, Vaud, Switzerland

We thank the reviewer for their response and positive feedback. We have addressed all comments as follow:

1. DNA barcoding was done to verify the species identity. This species is Europe's only representative of the Osmylus genus and is quite characteristic compared to other known neuropterans in both morphology and habitat.
2. We thank the reviewer for requesting clarification on the subsampling process. Subsampling from 164× to 60× coverage was performed using an in-house Python script that sequentially extracted PacBio HiFi reads until the target cumulative sequence yield was reached. Because PacBio FASTQ/FASTA files store reads in a random order following primary data processing, sequential iteration provides an unbiased, random selection of reads. No weighting or filtering based on read quality was applied; however, a length threshold was enforced to remove reads shorter than 1,000 bp prior to assembly. We now provide this information in the main genome report.
3. We thank the reviewer for requesting specific details regarding the Hi-C dataset quality, which we now provide in the main genome report. The primary issue with the library was not a high duplication rate—which was acceptable at ~25%—but rather a severe deficit in long-range genomic interactions. Specifically, intra-chromosomal (cis) read pairs providing structural linkage were exceptionally low: cis > 1 kb pairs: 3,886,877 (3.5%) and cis > 40 kb pairs: 2,890,649 (2.6%).
4. We have now reported the mitochondrial assembly in both Tables.

# Reviewer report: Reviewer response for version 2

**DOI:** 10.21956/openreseurope.26149.r76831

**Published:** 2026-07-28

**Authors:** Ian Carr

**Affiliations:** 1 University of Leeds, Leeds, UK

Regarding: ERGA-BGE chromosome-level genome assembly of the giant stream lacewing Osmylus fulvicephalus (Scopoli, 1763)

 The article describes the genome assembly of the giant stream lacewing and is succinct and easy to follow. The assembled genome appears to be of high quality. However, there are a few minor issues that need to be addressed.

 1) In the " **DNA/RNA processing"** section the sentence "Biobank barcode is FD20030308." appears. This is very short and lacks context. The authors expand on what sample they are referring to or possibly put this information in the "Vouchering information" section, which seems to cover biobanking.

 2) The authors direct the reader to the methods described on the PacBio website. Since this content is on a commercial website, I'm not sure how permanent it is, and the data may be moved or deleted; consequently, the authors need to provide a more permanent link to the methods or include it in the paper.

 3) In the main text the authors refer to the mitochondrial genome, but the organelles section of Table 1 is left blank; the table's organelle section needs to be filled in.

Are sufficient details of methods and materials provided to allow replication by others?

Partly

Is the rationale for creating the dataset(s) clearly described?

Yes

Are the datasets clearly presented in a useable and accessible format?

Yes

Are the protocols appropriate and is the work technically sound?

Yes

Reviewer Expertise:

NGS data analysis, rare recessive disease identification, eDNA, genome assembly

I confirm that I have read this submission and believe that I have an appropriate level of expertise to confirm that it is of an acceptable scientific standard.

# Response

**Published:** 2026-08-31

**Authors:** Chiara Bortoluzzi

**Affiliations:** Environmental Bioinformatics Group, Swiss Institute of Bioinformatics, Lausanne, Vaud, Switzerland

We thank the reviewer for their comment and positive feedback. We have addressed their comments as follow:

1. We now specified that the barcode belongs to the adult female used to extract the RNA.
2. We have removed the link to the PacBio website and described the essential parts of the DNA extraction method in more detail.
3. We now added the mitochondrial genome to both Table 1 and Table 2. 

# Reviewer report: Reviewer response for version 2

**DOI:** 10.21956/openreseurope.26149.r76833

**Published:** 2026-07-28

**Authors:** Fedor Čiampor

**Affiliations:** 1 Slovak Academy of Sciences, Bratislava, Slovakia

The data note presents a high-quality chromosome-level genome assembly for lacewing Osmylus fulvisephalus. The note is clearly structured and provides nice background on the species, including its distribution, life cycle, biology, and taxonomic placement. As far as I can judge, the methodology is detailed, well-described. The main contribution of the manuscript is the production of the high-quality genome for the O. fulvicephalus, which can contribute to clarifying the phylogenetic placement of the family *Osmylidae* within Neuroptera as well as to study evolution of their venom system.

 I have two small comments:

 In the Sample and sampling information - “following sex determination, specimens were flash-frozen in liquid nitrogen. No permits were required for sampling. Specimens were euthanised by being flash frozen in liquid nitrogen.” - I don't think it's necessary to repeat twice that the adults and the other individuals were frozen. One sentence is enough; it'll sound better that way.

 In the Vouchering information - “No physical reference material for the sequenced specimen has been deposited in a collection” - I don't quite understand this—it sounds as if these vouchers were thrown away. Were they really not set aside somewhere? If so, and they're not part of some older collection, it would be helpful to specify where they're being stored.

Are sufficient details of methods and materials provided to allow replication by others?

Yes

Is the rationale for creating the dataset(s) clearly described?

Yes

Are the datasets clearly presented in a useable and accessible format?

Yes

Are the protocols appropriate and is the work technically sound?

Yes

Reviewer Expertise:

invertebrate DNA (meta)barcoding

I confirm that I have read this submission and believe that I have an appropriate level of expertise to confirm that it is of an acceptable scientific standard.

# Response

**Published:** 2026-08-31

**Authors:** Chiara Bortoluzzi

**Affiliations:** Environmental Bioinformatics Group, Swiss Institute of Bioinformatics, Lausanne, Vaud, Switzerland

We thank the reviewer for their comment and positive feedback. We have addressed their comments as follow:

1. We rephrased the Sample and sampling information section to avoid any repetition.
2. We did not throw away any vouchers because they were simply not produced.

# Reviewer report: Reviewer response for version 2

**DOI:** 10.21956/openreseurope.26149.r76829

**Published:** 2026-07-20

**Authors:** Peng-Fei Ma

**Affiliations:** 1 Chinese Academy of Sciences, Kunming, China

In this study, the complete genome of the giant stream lacewing ( *Osmylus fulvicephalus*) was sequenced and assembled. More than 99% of the assembly was anchored onto 6 chromosomal pseudomolecules and the contig and scaffold N50 have a size of 51.5 Mb and 116.2 Mb, respectively. In short, this is a high-quality genome assembly, providing crucial genomic resource for studying the genetic basis of venom evolution in Neuroptera.   However, RNA extraction and sequencing were mentioned in the Materials and Methods while there are not associated results described in Results.

Are sufficient details of methods and materials provided to allow replication by others?

Yes

Is the rationale for creating the dataset(s) clearly described?

Yes

Are the datasets clearly presented in a useable and accessible format?

Yes

Are the protocols appropriate and is the work technically sound?

Yes

Reviewer Expertise:

Plant genomics and evolution

I confirm that I have read this submission and believe that I have an appropriate level of expertise to confirm that it is of an acceptable scientific standard.

# Response

**Published:** 2026-07-20

**Authors:** Chiara Bortoluzzi

**Affiliations:** Environmental Bioinformatics Group, Swiss Institute of Bioinformatics, Lausanne, Vaud, Switzerland

We thank the reviewer for their positive response and for accepting the publication of this genome report. We would like to clarify here that the RNA-Seq data produced are now being used by Ensembl to produce the genome annotation. As the genome annotation is ongoing, no results on the genome annotation are reported.

# Reviewer report: Reviewer response for version 1

**DOI:** 10.21956/openreseurope.25386.r73500

**Published:** 2026-05-09

**Authors:** Sivasankaran Kuppusamy

**Affiliations:** 1 Loyola College Chennai, Chennai, Tamilnadu, India

The title of the manuscript may be modified as *“*Chromosome-level genome assembly of giant stream lacewing *Osmylus fulvicephalus* (Scopoli, 1763)”.

 The section describing high molecular weight (HMW) DNA extraction would benefit from additional methodological detail. Please elaborate on the extraction protocol by including information such as the source tissue used, preservation conditions, extraction kit or reagents, incubation conditions, DNA purification steps, fragment size assessment, and quality/quantity evaluation methods.

 Genome assembly statistics are currently absent from the main text. It is recommended that a comprehensive assembly statistics table be included, containing details such as: Assembly level, Genome span (Mb), Number of chromosomes, Number of contigs, Contig N50, Number of scaffolds, Scaffold N50, Longest scaffold length (Mb), Identification of sex chromosomes and Organellar genomes assembled (mitochondrial)

 Annotation details of the genome assembly have not been sufficiently described in the manuscript. Please provide a summary of genome annotation results in the text, including the number of predicted protein-coding genes, non-coding RNAs, repeat content, and functional annotation statistics.

 In addition, chromosome pseudomolecules identified in the haplotype-resolved genome assembly of *Osmylus fulvicephalus* should be presented in a separate table, including chromosome identifiers, scaffold lengths, GC content, and associated assembly metrics where applicable.

 Overall, the manuscript was prepared well but needs revision.

Are sufficient details of methods and materials provided to allow replication by others?

Yes

Is the rationale for creating the dataset(s) clearly described?

Yes

Are the datasets clearly presented in a useable and accessible format?

Yes

Are the protocols appropriate and is the work technically sound?

Partly

Reviewer Expertise:

phylogenetic analysis of Noctuoidea moths using mitogenome sequence

I confirm that I have read this submission and believe that I have an appropriate level of expertise to confirm that it is of an acceptable scientific standard, however I have significant reservations, as outlined above.

# Response

**Published:** 2026-06-01

**Authors:** Chiara Bortoluzzi

**Affiliations:** Environmental Bioinformatics Group, Swiss Institute of Bioinformatics, Lausanne, Vaud, Switzerland

We thank the reviewer for their comments. We have addressed as follow:

1. We have slightly changed the title of the genome report to reflect the reviewer's suggestion
2. We have now provided some additional information in the section describing the HMW DNA extraction.
3. We now provide 2 Tables summarizing basic statistics concerning the genome assembly. Both tables are reported in the Results section.
4. We did not provide any annotation details because the genome annotation is currently undergoing by Ensembl. Once ready, the genome annotation will be published in the Ensembl website at this link: https://projects.ensembl.org/erga-bge.
