# Supplementary material for: ERGA-BGE reference genome of Osmylus fulvicephalus, the giant stream lacewing, Europe's representative of the family Osmylidae
Source: Open Res Eur. 2026 Apr 28;6:136. [Version 1] doi: 10.12688/openreseurope.23457.1 (PMC13554741; doi:10.12688/openreseurope.23457.1)
Supplement: Peer review history

# Response

**Published:** 2026-06-01

**Authors:** Chiara Bortoluzzi

**Affiliations:** Swiss Institute of Bioinformatics, Lausanne, Vaud, Switzerland

We thank the reviewer for their comments. We have addressed as follow:

1. We have slightly changed the title of the genome report to reflect the reviewer's suggestion
2. We have now provided some additional information in the section describing the HMW DNA extraction.
3. We now provide 2 Tables summarizing basic statistics concerning the genome assembly. Both tables are reported in the Results section.
4. We did not provide any annotation details because the genome annotation is currently undergoing by Ensembl. Once ready, the genome annotation will be published in the Ensembl website at this link: https://projects.ensembl.org/erga-bge.
